# Remarks on the Utility of Cathartics

**Published:** 1835

**Authors:** Lewellyn Powell

**Affiliations:** Louisville, Kentucky


### Art. II.—Cathartics.

*Remarks on the utility of Cathartics in several Diseases; designed to illustrate the leading principles which should regulate their use.* By Lewellyn Powell, M. D., of Louisville, Ky.

The very general and too often indiscriminate employment of purgative medicines, both in professional and popular practice, together with the discrepant opinions entertained in regard to the extent and manner of their therapeutic application, have contributed to this class of remedial agents, an amount of interest which belongs to none other; and establish their claims to a grave and careful consideration on the very broadest basis.

Assuredly there is no class of remedies, within the wide range of the materia medica, to which our inquiries can be more profitably directed; for there is none which so abundantly supplies to us appropriate and efficient means for combatting disease. Few are the cases, and more especially of acute disorder in which they can be altogether dispensed with, whilst into the treatment of very many they constantly and largely enter.

A strong persuasion of their salutary powers in the alleviation and cure of diseases, has obtained such prevalent ascendancy in the public mind, as well popular as professional, that in almost every form of malady incidental to our nature, cathartics are instantly and habitually resorted to, and in numberless instances without any regard to those guides and checks which the laws of the economy and the teachings of experience, have imperatively prescribed.

If such be the fact, with the obvious result that the unskilful use of purgative medicines has been productive of much positive evil, it cannot be an idle task, nor (even though imperfectly performed) without some useful tendency to consider the practical bearings of this subject in regular connexion and to an extent in some degree commensurate with its importance.

To the performance of this task I am now to proceed, with the humble ability I may possess, in a course of (as it seems to me at least) pertinent remarks on the direct or physical agency of cathartics; the general symptoms independent of specific disease which ordinarily call for their employment; the specific differences subsisting between the various cathartics in common use, and the general rules, founded in part on these individual peculiarities, which should govern their administration, and their fitness and application as a class in the cure of several diseases.

1. The peristaltic action of the intestines, by which is meant a regular and consecutive series of muscular contractions, propagated along the tube from above downwards, is the physiological movement by which the contents of the bowels are carried forward in their course, and with the aid of the abdominal muscles finally expelled.

It is principally by quickening these peristaltic motions, that purgatives produce their effect, but something is also to be allowed to the serous and mucous exhalations which follow their impression; and which, by softening the fœcal contents of the bowels, and lubricating the surface of the canal, diminish resistance to the propelling power, and greatly facilitate the evacuation designed to be produced.

2. Without the existence of specific or well defined disease, there are occasionally present, some decided indications, which authorize and call for the employment of cathartics. The chief of these are sluggish and costive bowels, with feeble, depraved or irregular appetite; a loaded, yellow or brown tongue, thirst with a vitiated or any unusual taste in the mouth; fulness of the lower belly with or without tenderness on pressure, and a turbid and bilious or saffron colored urine.

The general intentions designed to be fulfilled by the use of purgatives, whether called for by the circumstances just stated, or by the peculiar exigencies of any special disease are, 1st. To clear the intestinal canal. 2. To correct unhealthy secretions. 3d. To promote a free or copious discharge from the intestinal exhalents, with the view to reduce the mass of circulating fluid, and thus to lower excitement, and 4th. To lessen the determination of blood to particular parts.

3. While the whole class of cathartics agree in the general property of evacuating the intestinal canal, they are distinguished amongst themselves for modes of action and remote effects peculiar to each, and by which they are suited to the circumstances and indications which call for their use in the treatment of disease. For example, it is affirmed by writers on the Materia Medica, that calomel and gamboge expend their action chiefly on the upper portion of the intestinal tube; while colocynth and castor oil have a more extensive range of operation, and aloes exerts its cathartic power mainly upon the lower bowels. This remark, though to some extent true, is not to be received without qualification; thus I am disposed to deny that gamboge limits its operation to the upper portion of the intestine, or that its range of action is less extensive than that of colocynth or castor oil; while I am confident in the opinion, that calomel distributes its impression throughout the entire length of the canal, and that aloes too often excites and promotes the biliary discharge to allow of the conclusion, that the larger bowels only feel its influence.

Nor am I disposed to acknowledge the truth of a remark made by Dr. Eberle, that rhubarb and castor oil merely evacuate the ordinary fœcal contents of the bowels, for I have seen these articles excite a considerable amount of serous effusion into the intestines.

I make these remarks to show, that in my opinion, cathartics are less distinguished by the restriction or limitation of their action to different sections of the intestinal canal, than by their specific excitement of the different classes of secerning vessels which communicate with its cavity. Of these the biliary, the mucous, and the serous are the chief if not the only ones recognized in the present state of physiology; and cathartics differ remarkably and habitually in the degree in which they effect these different vessels, and in the amount of their respective secretions which they occasion to be discharged. Thus calomel specifically excites the liver and the mucous follicles of the bowels, throughout their entire length, producing bilious and consistent stools; and gamboge, colocynth, elaterium, croton oil, and some others stimulate the serous exhalents to extraordinary action, occasioning copious watery evacuations as the common and natural result of their employment; while some few, and such are aloes and rhubarb, when given in moderate doses, and not frequently repeated, merely quicken the muscular actions of the intestines, with the effect of discharging its fœcal contents unchanged and unmixed; but frequently repeated, even aloes and rhubarb, have a range of operation beyond this, and will sometimes produce bilious, serous or mucous discharges, according to the greater or less excitability of the different sets of vessels by which they are respectively furnished. This distinction which is founded in nature, and attested by extensive observation, is highly important, and has strong claims to our attention, because of its bearings upon practical medicine. Thus if it be the object of the practitioner, simply to unload the bowels of accumulated fœces, aloes and rhubarb are the articles best adapted to his purpose; if to quicken the portal circulation, to emulge the liver and thoroughly evacuate the bowels be his main design, then calomel and its adjuvants will claim his preference; while if the intention is to reduce a sthenic diathesis, to subdue arterial action, or to promote the absorption of serous effusions into the cavities or cellular tissue, jalap, elaterium, croton oil, and such others are alone adapted to the end in view.

Besides the objects already stated to be accomplished by the agency of cathartics, they frequently operate their best effects in the cure of disease, on the principle of revulsion, in conformity to that law of the animal economy, which determines that an unusual excitement and afflux in the vessels of any one part of the system, subtracts in both these respects from the vessels of certain other parts; and hence the undoubted utility of purgative medicines in diseases of the head and the skin.

These general considerations point to the necessity of a sound discrimination in the choice of purgatives, and call for the establishment of some general rules by which to regulate their administration, and to secure their appropriate and most beneficial action.

1st. We should ever select the purgative to be employed, with a nice adaptation of its peculiar powers to the special object contemplated in its use.

2d. If existing circumstances permit a choice of time, purgatives should be administered in the morning or at night, or on an empty stomach, by which they are better retained and more certainly and effectually operate, and for like reasons the period of remission or intermission should be selected in fever.

3d. Promote their free and easy operation by occasional draughts of warm gruel or chicken water, after the first discharge; and restrain their action when excessive by a resort to opiated clysters, and if necessary, to counter irritation by sinapisms to the surface.

4th. If drastic purgatives are employed, the harshness of their operation may be meliorated, by combining with them articles of a milder kind, without impairing the freedom and efficiency of the evacuations intended to be produced.

5th. In plethoric and robust subjects, when the bowels betray much insensibility to the impression of cathartics, recourse must be had to the lancet, the efficacy of which under these circumstances, in promoting the operation of purgative medicines, has long been observed.

The foregoing observations are essentially preliminary, and a necessary introduction to the chief design of this essay, which is to consider the use and value of purgatives in several of the diseases which most commonly demand the discipline of our art. Even when thus limited, the field of inquiry is sufficiently extensive and full of interest.

Among the therapeutic applications of cathartics, none is more frequent or of more decided utility, than to the management of fever.

To perceive their propriety in fever, as a leading measure of cure, we have but to advert to the early and constant derangements of the alimentary canal, which universally obtain in this most prevalent disease; and to reflect upon the important functions, the lively sympathies and extensive connexions which give to the intestines a conspicuous place, and a controling power amongst the organs of the human frame. Where is the case of idiopathic fever, in which the evacuation of the primæ viæ is not urgently demanded by the earliest and most prominent of those symptoms, which nature holds out as signals of her distress, and as instructive hints to the physician? The loathing of food, the coated tongue, the fœtid breath, the constipated or irritated bowels, the unnatural offensive and acrid stools, the nausea and vomiting, all afford the plainest indications for their employment, whilst the universal experience of enlightened practitioners attests their efficacy.

Of the power and utility of cathartic medicines in the cure of typhus, we were long since advised by the judicious Hamilton, whose work on purgatives, will ever merit the profoundest attention of practioners as a faithful record of his own valuable experience, and an instructive guide in the use of an active class of remedies; which if not understandingly employed, are quite as apt to prove a source of mischief as the means of good.

The manner in which Dr. Hamilton attained a knowledge of the remedial powers of cathartics in typhus fever, is interesting of itself, tending as it does to challenge confidence in his statements, and impress them upon the memory of the reader. He informs us, that when he was first appointed physician to the Royal Infirmary at Edinburg, it was customary in that institution to treat typhus fever with mild antimonials as the chief remedies, the main object held before the practioner being the removal of torpor and spasm of the extreme vessels supposed to exist, while after the onset of the fever, the bowels were wholly negected, or purgatives administered with distrust and apprehension, lest they should rivet the spasm and perpetuate the disease.

Disappointed in the results of this treatment, Dr. Hamilton wisely determined upon innovation, and gave to his patients a more active antimonial, and in larger doses than was usual in the practice of the Infirmary. The change was not without advantage; but not content with the notice of this general fact, he also discovered that the antimonial was beneficial in the cure of typhus just in proportion as it moved the belly; and hence was led to infer, that purgatives strictly so called, might usefully supersede the antimonials, with the great advantage of certainly attaining the purgative effect, without incurring the hazard of debility and exhaustion from violent vomiting or copious sweats.

A careful experience, extended and improved for the space of forty years, confirmed his conjectures and enabled him to declare with much certainty in regard to the treatment of typhus fever, that the full and regular evacuation of the bowels relieves the oppression of the stomach, cleans the loaded and parched tongue, mitigates thirst, restlessness and heat of surface, prevents the alarming symptoms too commonly developed in the advanced stage, more certainly and speedily promotes recovery, and diminishes the danger of relapse.

This sound practioner refers the superior utility of purgatives in typhus, to their operating throughout the whole extent of the intestinal canal, and acting directly upon an organ, the healthy functions of which are essential to recovery, in a manner that is consonant to the course of nature by propelling its contents downwards, and moving and completely evacuating the feculent matter, which from detention and diseased secretion has become highly offensive and hurtful.

There is reason to believe, that many amongst the readers of Hamilton, under a misapprehension of his views, have greatly abused this valuable class of medicines, and by the too frequent and very prodigal use of drastic cathartics, have rather aggravated than meliorated the disease; and have tended to bring into disrepute a course of practice, which under the limitations assigned by Hamilton himself, is entitled to our very highest respect, and is susceptible under judicious control of the most beneficial application.

While he strictly enjoins the daily and complete evacuation of the bowels throughout the whole course of fever, from its beginning to its termination, Dr. Hamilton expressly dedeclares, that he is no advocate for exciting unusual secretion into the cavity of the intestines, and procuring copious watery stools, which make no salutary impression on the disease, and may alarmingly increase the debility of the patient.

The general principles thus perspicuously stated in the work of Hamilton, are so well illustrated and sustained by his clinical reports, that it is with peculiar satisfaction that I notice his able demonstration of a truth that bears with such weighty import upon the practice of our art.

Armstrong has also given to cathartics a prominent place in the treatment of typhus; and in his standard and classical work, we are furnished with the most judicious instructions as to the time and manner of their employment. It has been clearly demonstrated by these eminent writers, and abundantly proved in the common experience of practitioners, that the debility so strikingly characteristic of typhus, and which is generally regarded with so much apprehension, can only be meliorated and removed by unloading the system by evacuants, and that none are so safe and effectual as cathartics. It is, however, too frequently and fatally forgotten, that it is only through the earlier and excited stages of typhus, that active or frequent purging can be safely employed.

It cannot be too deeply impressed upon the mind of the physician, that when the aspect of the case betrays the approach of collapse, purgatives must either be substituted by enamata, or cautiously administered in very reduced doses, while the waning strength is at the same time sustained by diffusible stimuli of such kind and in such quantity, as the rate of declension in the general powers may seem to demand in each epidemic or every individual case.

In the autumnal fevers of our own country, whether of intermittent, remittent or continued type, cathartic medicines are of indispensable utility. It was the custom of Cleghorn, whose writings display an intimate acquaintance with tertian fevers, as well as much practical sagacity, after premising blood-letting at the height or towards the decline of the paroxysm, to make use of the first remission or intermission, to evacuate the first passages, for (he remarks) the disagreeable taste in the mouth, loathing of food, giddiness, pain in the forehead, and loins, and other constant attendants of tertian fever, make it evident that the stomach and intestines are loaded with vitiated secretions, particularly morbid bile, and which if not early discharged, will induce very threatening symptoms in the course of the fever, such as violent vomiting, redoubling or continuation of the paroxysms, restlessness, ravings, pain, inflammation, gangrene of the small intestines, and lastly, sudden death.

To mitigate and prevent these distressing symptoms, much is accomplished by the daily use of cathartics, for the four or five first days of the disease. But an error is committed, and in the malarious districts of the south, too often a fatal error, in relying too long and too exclusively upon purgatives for the cure of intermittents. In discharging the vitiated feculence, and the morbid secretions which collect in the intestinal canal, cathartics perform a very necessary and salutary part in the treatment of these fevers; but they will not always prevent the recurrence of the paroxysm, which is far the most important point of treatment, in the insidious and malignant forms of tertian, which occasionally prevail in high latitudes and hot seasons. To guard against those dangerous revolutions, which stealthily, or with sudden violence, wreck and overwhelm the system, we can look with confidence only to the cinchona, or its active principle, the quinine, whose powers and application in malignant tertians have been so fully exhibited by Cleghorn and Alibert, and will satisfactorily appear to all who will resort to the sure test of experience.

In our bilious remittent or continued fevers, cathartics are equally considered as of indispensable utility: and no practitioner, who is untrammelled by the dogmas of Broussais, would attempt the cure of these fevers without the aid of purgatives, any more than he would treat pleurisy or pneumonia without a recource to the lancet or antimony.

From Sydenham to Pringle, from Pringle to Rush, and from his day to our own, the most distinguished writers and the ablest practitioners have borne such ample and decided testimony to their suitableness and efficacy, in remittent and continued bilious fevers, that these points may now be considered as beyond all doubt or cavil.

Dr. Rush's history of the epidemic of 1793, is replete with proof and instruction in regard to the power and use of purgatives in the fevers of this fami'y. And it is curious and gratifying to observe, how immediate and striking was the improved success of his practice in yellow fever, after he had laid aside the unsettled and discrepant methods of treatment then in vogue, and placed his confidence in mercurial purges.

But it is not enough for us to know the general fact, that purgatives are useful remedies in remittent fevers: It is equally important that we possess precise information as to the selection and administration of the purgatives best suited to the ends in view.

Cleghorn thought Epsom salts sufficient for the general purposes of this class of medicines, in the treatment of fever; and there is no doubt that in the milder forms and earlier stages of the disease, it will oftentimes supersede the necessity of a resort to more active means. But the vast majority of experienced physicians concur in recommending the mercurial purges; and by common consent calomel is made the leading ingredient in the cathartic compounds usually prescribed for the whole tribe of autumnal fevers.

Both because there is a continual reproduction of morbid bile, and because we are required to repeat our efforts as long as the disease persists, it is necessary to administer the purgatives every day to the extent of procuring from four to six free evacuations, of a dark, consistent, and fœtid material, the expulsion of which, it is agreed by all, is very generally attended with an abatement of the disease. I am here disposed to depart from an opinion offered by Dr. Chapman, that "this peculiar matter is a product of diseased action, and previously existed in the intestines, in close adhesion to their surface;" nor do I regard it, with him, as a maintaining cause of fever, and on this account deem it necessary to expel it. But on the contrary I hold it to be a peculiar secretion chiefly mucous, but more or less mixed with bile, *specifically* excited by the mercurial compounds administered, and tending more than any other kind of discharge to deplete and unload the excited or congested vessels and glands mainly involved in the cause and burden of the disease. I would insist upon this point as one of much practical importance, for it is somewhat concerned in regulating the use of mercurial purges in the treatment of fever. Under the view of Dr. Chapman it has been stated as a rule of practice, to purge, and with calomel, until the alvine evacuations become of a healthy and ordinary bilious complexion; and accordingly, in the eager pursuit of this object, practitioners have confined their attention so exclusively to the intestinal secretions, as to overlook other circumstances, which no less certainly inform us of the progress of the disease, and have pushed their remedies beyond the point, where they might reasonably and with advantage be suspended or give place to others. In my apprehension of the subject, the alvine secretions are of subordinate relation and secondary importance to the condition of the nervous and circulating systems; and we shall find, in a course of careful observation, that not unfrequently, when the subdued irritation of the nervous, and the equal action of of the vascular system, indicate the rapid decline of disease, and the near approach of health, the dejections from the bowels are of the same general appearance, (calomel being administered,) as at the height of the disease; and the pertinacious attempt to accomplish the desired change, through the agency of mercurial purges, will too often result in the supervention of mercurial fever, painful salivation, broken health, and permanent infirmities of constitution.

Appropriate and valuable as purgative medicines unquestionably are in most forms and cases of fever, yet we must not lose sight of the very important fact, that *inflammation* of the mucous membrane of the stomach and small intestines, though not the origin and essence of the disease, as assumed by the great French Pathologist, is nevertheless a frequent complication; and when we encounter cases of fever thus complicated, as characterized by vomiting, purging of thin matter, tender epigastrium, white or brown tongue, with red edges and disposed to dryness, much thirst, contracted pulse, and anxious countenance, it would be a departure from sound practice, and an abuse of medicine, to attempt their cure by a course of drastic or active purgatives.

It is under such circumstances that we may admit the force of Broussais' objections, who in pointing out these exceptions to the use and efficacy of purgatives in the cure of fevers, has entitled himself in a very considerable degree to be considered a reformer in medicine. Far better is it in cases of this description, to rely upon leeches and ptysans, than to trust to the ultra purgative plan, which is indiscriminately and unrelentingly pursued from first to last, with no definite object but a change in the stools and with no restrictions save ptyalism or death.

The utility of cathartics is by no means limited to the idiopathic fevers. They also hold a prominent and important place in the treatment of eruptive fevers. To some extent, the use of purgatives is demanded in the exanthemata, upon the same principles that call for their employment in ordinary remittent and continued fevers. In the former as in the latter, the primæ viæ are to be cleansed of their vitiated contents the force of arterial action is to be subdued and congestions of the brain and liver are to be prevented or removed.

But in the exanthematous fevers, there is another consideration which makes an additional requirement for the use of purgatives. The morbid action of the cutaneous capillaries may, and in many instances does, reach to such excess, that by reason of their sympathetic relations, excitement, or derangement of some kind is communicated to other important organs. Accordingly, in small pox where to lessen the quantity of eruption is an indication of acknowledged importance, purgatives could not fail to be suitable and useful remedies, since by inviting excitement and afflux to the bowels, they relieve the skin of that excess of morbid action which is otherwise, and injuriously reflected upon other organs. Hence it is that, ever since the illustrious Sydenham established the fitness of antiphlogistic treatment in small pox, the use and efficacy of purgatives in this disease have been fully recognized.

With such modification as their respective circumstances will readily suggest, the foregoing remarks will equally apply to measles and erysipelas, in both of which diseases cathartic medicines are habitually and beneficially employed.

Cathartics have been variously regarded by physicians, in reference to scarlatina. By some they are prescribed with diffidence, and denounced by others, while by a third, and I would hope the most numerous class, they are esteemed most appropriate and valuable remedies in this formidable disease. To Hamilton belongs the credit of having weakened the prejudice, which at one time extensively prevailed against the use of purgatives in scarlitina. He rightly apprehended that the debility which is conspicuously present in this fever, and upon which alone was founded the distrust and dread of purgatives, did not more properly than in typhus contra-indicate their use; and on the contrary, in the one case as in the other, there was all reasonable ground to expect that the debility would be diminished, rather than increased under their operation. Directed by such such views he made common and extensive use of purgatives in the cure of scarlatina, and declares with much earnestness, that he never, in a long course of experience, witnessed sinking and fainting, as mentioned by some authors, and so much dreaded by them; neither had he observed revulsion from the surface of the body, and consequent premature fading, or, in common language, striking in of the efflorescence from the exhibition of purgatives; hence he employed them with confidence, no variety of the disease, as appearing in different epidemics, or in the course of the same epidemic, deterred him from following out this practice to such extent as he deemed necessary; and by which he was generally enabled to mitigate and remove the pungent heat of surface, the violent head ache, the turgescence of the features, the flushing of the countenance, and the full and quick pulse.

Armstrong even to a greater extent is also the advocate of purgatives in the treatment of scarlatina; and I may safely declare that I have accomplished more, in the cure of this fearful malady, by the use of purgatives, when opportunely given, and regular and kindly evacuations could be procured, than by any other class of remedies.

The observations of Hamilton, unless perused carefully and with reflection, are in some degree calculated to mislead the reader; when this excellent physician remarks, that he never witnessed sinking and fainting from the exhibition of purgatives, nor observed the premature fading or striking in of the efflorescence, he certainly does not mean to affirm, that any kind and degree of purging can be practiced in scarlatina without danger of such consequences, for such an assertion would be contradicted by the experience of every practitioner who is at all conversant with the disease. Neither does he intimate, that no restraints are to be imposed upon the use of purgatives, when he tells us that in no variety of the disease, whether of different epidemics, or in the same epidemic, was he deterred from following out this practice, to the extent which he found necessary. Hamilton never witnessed sinking and fainting from the exhibition of purgatives, in his own practice, because he carefully confined their exhibition within the limits of moderation and prudence. He was content to give one or two brisk purgatives in the beginning, and avoided full purging in the subsequent periods of the disease, where, in his judgment, the only proper object was to "remedy the impaired action of the intestines, to secure the regular expulsion of their contents, and thus to prevent the accumulation of fæces, which never fails to aggravate the symptoms, and to prove the source of further suffering to the patient." And he was not deterred from following out the practice to the extent which he deemed necessary, because this extent was regulated by a judicious choice of purgatives, and these skilfully adapted in their power, quantity, and mode of combination, to the various circumstances distinguishing the different epidemics and individual cases.

But however valuable purgatives are in the general treatment of scarlatina, emetics are justly entitled to take precedence of them, in the earlier stages of the disease, when marked by strong expressions of depression, and loss of balance in the nervous system, with a delayed, imperfect, or retrocent eruption. In determining to the surface, and disposing to evacuation by the skin, emetics would seem, more than any other remedies, to conform in their operation, to the natural tendency of this disease; which runs a determinate course, through a series of mysterious processes, that terminate in a peculiar and concentrated action of the skin; and on the developement of which there occurs a solution or suspension of the morbid movements.

Dr. Chapman well remarks, that in this disease "Nature works to the deliverance of the system," and it may be added, that she works under the direction of laws that may not be thwarted with impunity by the officious and ill-judged interference of Art. It is in such cases that the "nimia diligentia Medici" is too often conspicuously and mischeviously displayed, and where the practitioner, losing sight of the special nature and established tendencies of the disease, prematurely and inconsiderately bleeds and purges his patient to such a reduction of his constitutional energies, that the system is unable to develope the natural crisis of the disorder, and the morbid actions, as in the metastasis of gout and rheumatism, take an unnatural direction, and run on to an unfortunate issue.

It is thus that regular bred physicians, too exclusively occupied with the inflammatory nature of exanthematous fevers, have sometimes treated them with much less success than has attended upon domestic and popular practice. The experience of almost every epidemic, has furnished examples thus humbling the pride of science. It is but a few years since a populous and wealthy valley in Virginia, was extensively ravaged by scarlatina, in spite of the lancet and calomel, with which it was encountered; whilst among the pauper population of the mountainous district which surrounded it, the epidemic was rendered comparatively harmless, by the diffusible stimulants and diaphoretic drinks, to which these simple people were led by the unbiassed observation of nature.

But here, let me not be misunderstood, it is very far from my intention, to recommend the indiscriminate use of emetics or stimulants in the treatment of scarlatina, or to detract, in any degree, from the fitness and value of blood-letting and purgatives, as remedies in this formidable affection; but my object is to insist, that while both classes of remedies may be necessary in different cases, and in different stages of the same case, it better accords with the pathology of the disease, to treat it in its incipient stage, before the eruption is established, with gentle emetics, aided by the warm bath and diaphoretic drinks, than to have an early resort to blood-letting and purgatives, eminently useful and indispensably necessary as the latter are when re-action has been fully developed, and more decidedly still, when vehemently expressed.

No fact is better established, than that the danger of scarlet fever is connected with two different conditions of the vital organs, viz. congestion and inflammation: the former dependant on a depressed state of the nervous system, and characterized by pale surface, small pulse, great lassitude, and black blood; the other associated with an excited state of the nervous system, attended with high vascular action, and obvious tendency to inflammation. Not only do these internal congestions exist in the inceptive stage of the exanthematous fevers, and plainly indicate the propriety of such remedies as distribute and equalize excitement and circulation, but these congestions are liable to be fixed and quickened into inflammation, by the operation of that mysterious and unexplained property, (metastasis,) which forms part of the intimate nature of these diseases, whenever their tendency to a final and critical action by the skin is interrupted by a strong counteracting influence. By the tenor of these remarks, I do not mean to manifest any inclination to the errors of humoralism, nor to speculate upon purely hypothetical grounds; but I wish to represent, broadly and strongly, the necessity of regulating our remedial efforts in this class of diseases, with a due reference to a pathological principle, whose existence is acknowledged by the most eminent of modern authorities, and demonstrated by numerous facts, familiar to the experienced practitioner. Thus Professor Conolly, of the London University, in the course of some very judicious remarks, in illustration of several important points of pathology, observes, that "new directions given to certain portions of the blood, together probably with a transference of morbid actions in the nerves, may cause not only the conversion of gout to mania, but also of erysipelas of the scalp to phrenitis, or of erysipelas of the trunk to subinflammation of the mucous membrane of the intestinal canal; the sudden repulsion of some of the disorders of the scalp in children, as porrigo, is not unfrequently followed by meningitis. And the sudden recession of the cutaneous efflorescence in scarlatina, measles, and small pox, is commonly followed by inflammation of the lungs, or bowels, or brain.

For the sake of being explicit in a matter of such nice and critical concern, as the application of cathartics to the cure of exanthematous fevers, I have urged these general reflections, even to prolixity, and have swelled this essay so far beyond the limits which I had assigned it, as to preclude the remarks which it was my intention to have extended to the relationship between cathartic medicines and the bowel complaints, more especially cholera and dysentery. I forbear, however, to trespass longer upon the patience of my reader, and shall finish my task with expressing the hope, that the effort, feeble and imperfect as it is, may at least contribute to awaken attention to so extensive and important a subject as I have attempted to discuss, and which merits investigation for its own sake.

*Louisville, Kentucky, March,* 1835.

